# Targeting EGFR/PI3K/AKT/mTOR Signaling in Hepatocellular Carcinoma

**DOI:** 10.3390/pharmaceutics15082130

**Published:** 2023-08-14

**Authors:** Jieun Bang, Mihyeon Jun, Soyun Lee, Hyuk Moon, Simon Weonsang Ro

**Affiliations:** Department of Genetics and Biotechnology, College of Life Sciences, Kyung Hee University, Yongin-si 17104, Republic of Korea; qkdwldms612@khu.ac.kr (J.B.); mihyeonjun@khu.ac.kr (M.J.); lsoyun15@khu.ac.kr (S.L.); hmoon@khu.ac.kr (H.M.)

**Keywords:** hepatocellular carcinoma, EGFR/PI3K/AKT/mTOR signaling, animal models, targeted therapy

## Abstract

Hepatocellular carcinoma (HCC) poses a significant global health concern, with its incidence steadily increasing. The development of HCC is a multifaceted, multi-step process involving alterations in various signaling cascades. In recent years, significant progress has been made in understanding the molecular signaling pathways that play central roles in hepatocarcinogenesis. In particular, the EGFR/PI3K/AKT/mTOR signaling pathway in HCC has garnered renewed attention from both basic and clinical researchers. Preclinical studies in vitro and in vivo have shown the effectiveness of targeting the key components of this signaling pathway in human HCC cells. Thus, targeting these signaling pathways with small molecule inhibitors holds promise as a potential therapeutic option for patients with HCC. In this review, we explore recent advancements in understanding the role of the EGFR/PI3K/AKT/mTOR signaling pathway in HCC and assess the effectiveness of targeting this signaling cascade as a potential strategy for HCC therapy based on preclinical studies.

## 1. Introduction

Liver cancer presents a significant global health challenge due to its rising incidence and mortality rates. According to the World Health Organization, liver cancer accounted for approximately 800,000 deaths, positioning it as the fourth leading cause of cancer-related mortality [1]. Liver cancer comprises a heterogeneous group of malignant tumors with varying histologic characteristics and poor prognoses. Among these, hepatocellular carcinoma (HCC) represents the most prevalent primary liver cancer, accounting for approximately 80% of cases, followed by cholangiocarcinoma (CC), which contributes to 10–20% of primary liver cancers [2,3].

Existing treatment modalities for HCC offer limited success, with a considerably low 5-year survival rate. Surgical resection or local ablation therapy is typically favored for early-stage HCC; however, tumor recurrence occurs in approximately 70% of patients within 5 years [1,4]. For advanced HCC, systemic therapy is recommended as the standard treatment option, but the overall prognosis has been unsatisfactory [1,2].

HCC is a heterogeneous tumor with diverse phenotypic and genetic characteristics, and its tumorigenesis involves a range of molecular mechanisms [5,6]. Extensive research on the molecular pathogenesis of HCC has identified several critical signaling pathways involved in tumor initiation, promotion, and metastasis. These pathways include the mitogen-activated protein kinase/extracellular signal-regulated kinase (MAPK/ERK), Wnt/β-catenin, Hedgehog (HH), Hippo-YAP/TAZ, and phosphatidylinositol 3-kinase /AKT/mammalian target of rapamycin (PI3K/AKT/mTOR) signaling pathways. Recently, we have extensively reviewed the roles of MAPK/ERK, Wnt/β-catenin, HH, and Hippo-YAP/TAZ signaling in HCC development together with preclinical and clinical studies targeting signaling pathways in HCC [7,8].

In this review, our aim is to provide an overview of the tumor-promoting effects exerted by the PI3K/AKT/mTOR signaling pathway in hepatocellular carcinoma (HCC), which is primarily triggered by the epidermal growth factor receptor (EGFR). Additionally, we discuss the results from recent preclinical and clinical studies that target the EGFR/PI3K/AKT/mTOR signaling cascade as a potential therapeutic approach for treating HCC.

## 2. Role of EGFR/PI3K/AKT/mTOR Signaling Pathway in HCC

### 2.1. Overview of EGFR/PI3K/AKT/mTOR Signaling Pathway

**Figure 1:**
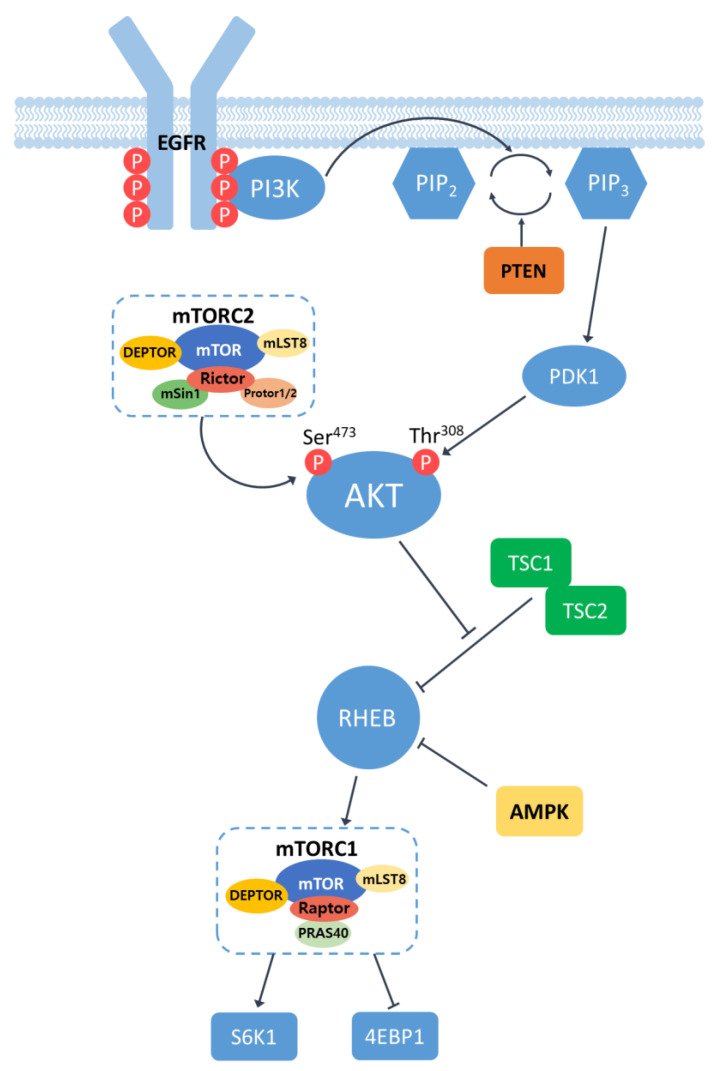
Schematic illustration of EGFR/PI3K/AKT/mTOR signaling pathway. Receptor dimerization induces the phosphorylation of tyrosine residues at the c-terminal cytoplasmic domain of EGFR. PI3K binds to phosphorylated tyrosine residues on EGFR and converts phosphatidylinositol-4,5-disphosphate (PIP2) in the plasma membrane into phosphatidylinositol-3,4,5-trisphosphate (PIP3), leading to the recruitment and subsequent phosphorylation of AKT by PDK1 and mTORC2. Phosphorylated AKT, in turn, leads to the activation of mTORC1 by regulating the activity of RHEB. Active mTOR induces the phosphorylation and activation of S6K1 and 4EBP1.

Epidermal Growth Factor Receptor (EGFR) is a cell surface receptor that belongs to the ErbB family of receptor tyrosine kinases. EGFR is activated by binding specific ligands, such as the epidermal growth factor (EGF) or transforming growth factor-alpha (TGF-α). Ligand binding induces receptor dimerization, leading to the activation of the intrinsic kinase activity of EGFR. The catalytic domain of EGFR kinases carries out a transphosphorylation process by which an array of tyrosine residues at the c-terminal cytoplasmic domain of EGFR are phosphorylated by its dimerized partner. Phosphotyrosine (pY) at the cytoplasmic domain of an activated EGFR acts as a docking site for proteins containing Src homology 2 (SH2) domains, such as Phosphatidylinositol 3-kinase (PI3K). The SH2 domain of PI3K binds to phosphorylated tyrosine residues on EGFR, bringing PI3K in close proximity to the plasma membrane. Upon recruitment, PI3K converts phosphatidylinositol-4,5-disphosphate (PIP2) in the plasma membrane into phosphatidylinositol-3,4,5-trisphosphate (PIP3) [9,10]. This conversion is carried out by the lipid kinase activity of the catalytic subunit of PI3K. The conversion of PIP2 to PIP3 can be antagonized by lipid phosphatase PTEN that converts PIP3 into PIP2 [11]. PIP3 serves as a docking site for proteins containing pleckstrin homology (PH) domains, such as the serine/threonine kinase AKT (also known as protein kinase B). Thus, the presence of PH domains in AKT allows the serine/threonine kinase to be recruited to the plasma membrane where AKT is phosphorylated at two critical residues: Thr308 by phosphoinositide-dependent kinase 1 (PDK1) and Ser473 by the mammalian target of rapamycin complex 2 (mTORC2). These phosphorylation events lead to the full activation of AKT [12]. Fully activated AKT phosphorylates regulates numerous downstream effectors, including proteins involved in cell survival, protein synthesis, metabolism, and cell-cycle progression. One of the key targets of activated AKT is the mammalian target of rapamycin (mTOR), which exists in two complexes: mTORC1 and mTORC2 (Figure 1). 

The mammalian target of rapamycin (mTOR) is a serine/ threonine protein kinase that regulates tumor growth, proliferation, metabolism, cell growth, and immunity [13]. mTOR has two complexes, which are mTORC1 and mTORC2. mTORC1 consists of mTOR, which is a regulator-associated protein of mTOR (Raptor), the DEP domain-containing mTOR interacting protein (DEPTOR), mammalian lethal with SEC13 protein 8 (mLST8), and the proline-rich AKT substrate 40 (PRAS40). mTORC2 contains the rapamycin-insensitive companion of mTOR (Rictor), DEPTOR, mLST8, Rictor-1 (Protor-1), Protor-2, and mammalian stress-activated protein kinase-interacting protein 1 (mSin1). mTORC1 is involved in cell growth and proliferation through nutritional sensing, and mTORC2 regulates the rebuilding of cytoskeletons and cell survival [14]. To activate mTORC1, RHEB, a small GTPase, is required. However, RHEB is usually inactivated by TSC1/2, a GTPase-activating protein (GAP) inhibitor [15]. The activated AKT can inhibit TSC1/2 and let RHEB be activated, resulting in the activation of mTORC1 and its downstream targets. Active mTORC1 increases protein synthesis by phosphorylating the eukaryotic translation inhibition factor 4E binding protein1 (4EBP1) and ribosomal proteins S6 kinase 1/2 (S6K1/2). Considering that these cellular processes are closely involved in carcinogenesis, it is no surprise that the dysregulation of the mTOR pathway has been observed in multiple human solid tumors [16].

### 2.2. Activation of EGFR/PI3K/AKT/mTOR Signaling Pathway in HCC

The EGFR/PI3K/AKT/mTOR signaling pathway is a major pathway in diverse types of cancers. This pathway actively regulates various aspects of cellular processes, including cell proliferation, survival, migration, and metabolism. It also plays a pivotal role in regulating the tumor microenvironment via angiogenesis and the recruitment of inflammatory cells [17]. Also, the EGFR/PI3K/AKT/mTOR signaling is significantly involved in therapy response and metastasis [18,19]. Considering the multifaceted roles in carcinogenesis, it is no surprise that the signaling pathway is commonly upregulated in a variety of human cancers [20]. 

In HCC, the EGFR/PI3K/AKT/mTOR pathway is abnormally activated in approximately 50% of cases, and this dysregulated activation is involved in various cellular processes, including cell proliferation, tumor cell differentiation, autophagy, metabolism, and the epithelial–mesenchymal transition (EMT) [14]. EGFR overexpression occurs in 68% of human HCC cases and significantly correlates with metastasis, poor patient survival, and aggressive tumors [21]. Moreover, EGFR ligands are overexpressed in human liver cancer cells and tumor tissues [22]. In higher stages of HCC, this signaling pathway is associated with vascular invasion, poor differentiation, and reduced survival rates [23]. Additionally, it has been reported that 40% of HCC patients who underwent orthotopic liver transplantation exhibited elevated mTOR activity [24].

PTEN suppresses AKT activation by converting PIP3 to PIP2. The loss of tumor suppressor PTEN and reduced expression of PTEN are primarily observed in the majority of patients with HCC in the early stages [25,26]. Somatic mutations in PTEN have been reported in HCC tissues [27,28]. Moreover, Fujiwara et al. observed 12 allelic losses of PTEN in 37 patients with HCC [29]. Loss-of-function mutations in TSC1/2 were found in approximately 20% of patients with HCC, which serves as another major suppressor of the EGFR/PI3K/AKT/mTOR signaling pathway [30].

## 3. In Vitro Studies Investigating EGFR/PI3K/AKT/mTOR Signaling in HCC Cell Lines

### 3.1. Tumorigenic Roles of EGFR/PI3K/AKT/mTOR Signaling in HCC Cells 

The activation of the EGFR/PI3K/AKT/mTOR pathway has been observed in various cancer types. Although ligand binding to EGFR is the natural process, which induces the dimerization of the receptors and subsequent phosphorylation of the cytoplasmic tails, the overexpression of EGFR alone can lead to the enhanced formation of its dimerization and activation of the downstream signaling pathway in the absence of its ligands. EGFR overexpression can be achieved through various mechanisms [31]. In addition to gene amplification and the epigenetic upregulation of the EGFR gene, changes in positive or negative regulators of EGFR can also affect its abundance in cancer cells. For example, NT5DC2 suppresses the ubiquitination of EGFR, preventing its ubiquitin-mediated proteasome degradation and leading to increased EGFR levels. In HCC cell lines, such as MHCC97H and PLC/RLF/5, the upregulated NT5DC2-induced overexpression of EGFR and the activation of the downstream PI3K/AKT/mTOR signaling pathway occurs [32]. Similarly, Song et al. reported that 14-3-3σ can interact with EGFR and stabilize the receptor, prolonging the activation of EGFR signaling in Huh7 and HepG2 cells [33]. Tropomodulin 3 (TMOD3), a member of the pointed-end capping protein family, is significantly upregulated in HCCs and is correlated with poor survival in patients with HCC. In various HCC cell lines, including Huh-7 and Hep3B, TMOD3 was found to facilitate the phosphorylation of the cytoplasmic tail of EGFR, triggering the activation of the downstream PI3K/AKT/mTOR signaling cascade [34].

In addition to the EGF/EGFR-mediated activation of the PI3K/AKT/mTOR signaling pathway, various molecules can contribute to eliciting this pathway. Recently, DEAD/DEAH box helicase 11 (DDX11) and Apelin (APLN) were identified as activators of the PI3K/AKT/mTOR signaling pathway in HCC. The overexpression and/or knockdown of these molecules substantially altered the PI3K/AKT/mTOR signaling in HCC cell lines and significantly affected the tumorigenic potentials of HCC cells [35,36]. Alpha-fetoprotein (AFP), which is strongly correlated with the aggressiveness of HCC, is a serum biomarker routinely used for the diagnosis and prognosis of HCC. Recently, Wang et al. investigated the role of AFP in HCC using two HCC cell lines. They reported that AFP interacted with PTEN, a negative regulator of AKT, and activated the PI3K/AKT/mTOR signaling pathway [37]. The role of claudin-6 (CLDN6) was investigated in HepG2 cells, where CLDN6 was found to activate the EGFR/PI3K/AKT/mTOR signaling pathway. The knockdown of CLDN6 led to decreases in the proliferation, migration, and invasion of HepG2 cells [38]. MicroRNAs (miRNAs) can also contribute to the activation of the EGFR/PI3K/AKT/mTOR signaling pathway. For example, miR-494-3p is correlated with aggressive clinicopathological characteristics and poor prognosis in HCC patients. Lin et al. showed that miR-494-3p bound to the 3′UTR of PTEN mRNA and repressed its translation in two HCC cell lines, SMMC7721 and HCCLM3 [39]. Notably, the suppression of PTEN expression by the ectopic expression of miR-494-3p led to the activation of the PI3K/AKT/mTOR pathway and enhanced the metastasis potentials of HCC cells [39]. Another in vitro study using the PLC/PRF/5 HCC cell line showed that the Mac-2-binding protein glycan isomer (M2BPGi) activated mTOR and exerted tumor-promoting effects on HCC [40].

### 3.2. Anti-Tumor Effects of Targeting EGFR/PI3K/AKT/mTOR Pathway in HCC Cells

Given that the EGFR/PI3K/AKT/mTOR signaling pathway exerts strong tumor-promoting effects on HCC, it is reasonable to attempt to inhibit this pathway for the treatment of HCC. EGFR is a direct target of miRNA-133b. The overexpression of miRNA-133b significantly suppressed EGFR protein expression and led to decreased activities of PI3K, AKT, and mTOR in the HepG2 cells [41]. Of note, the inhibition of the EGFR/PI3K/AKT/mTOR pathway via the overexpression of miRNA-133b induced the activation of caspase-3/-8 and apoptotic cell deaths in HepG2 cells. Methyltransferase like 14 (METTL14) can destabilize EGFR mRNA via an N6-methyladenosine (m6A) RNA methylation. METTL14 is significantly downregulated in HCC and is associated with the poor prognosis of HCC patients [42]. Similarly, an m6A-binding protein, YTH-Domain Family Member 2 (YTHDF2), binds to m6A sites in 3’UTR of EGFR mRNA and promotes the degradation of EGFR mRNA in HCC cells. In HCC cells such as HEP3B and SMMC7721, the overexpression of YTHDF2 suppressed cell proliferation via destabilizing EGFR mRNA and, thus, acting as a tumor suppressor [43]. In summary, inhibiting EGFR by the overexpression of endogenous EGFR suppressors such as miRNA-133b, METTL14, and YTHDF2 led to the subsequent inactivation of the downstream PI3K/AKT/mTOR signaling pathway and effectively reduced the malignancy of HCC cells by inducing cell apoptosis and suppressing cell proliferation.

In line with the observations in HCC cells characterized by the overexpression of endogenous suppressors of EGFR, the pharmacological inhibition of EGFR in HCC cell lines elicited similar effects. Specifically, it led to the suppression of cell proliferation and the induction of apoptosis in HCC cells through the downregulation of PI3K, p-AKT, and p-mTOR. For instance, the treatment of HepG2 cells with GW2974, an EGFR inhibitor, induced the attenuation of the downstream PI3K/AKT/mTOR signaling pathway, resulting in decreased cell proliferation and increased apoptosis in HCC cells [41]. Moreover, the inhibition of EGFR using erlotinib demonstrated inhibitory effects on the migratory capabilities and cell proliferation of HCC cells [32,44]. Similarly, the administration of EGFR inhibitors AG1478 and Gefitinib led to a reduction in cell proliferation, decreased invasion, and enhanced apoptosis in HCC cells [38,45].

In addition to targeting EGFR, the inhibition of its downstream effectors, such as PI3K, AKT, and/or mTOR, exerted similar tumor-suppressing effects on HCC cells (Table 1). Its treatment with LY294002 or Wortmannin, potent inhibitors of PI3K, downregulated the phosphorylated levels of AKT and induced apoptosis in HCC cells such as MHCC97 and Huh7 [46]. The treatment of HCC cells with a PI3K inhibitor 740Y-P showed similar effects [47]. These results consistently show that the inhibition of PI3K leads to the suppression of tumor growth and the induction of apoptosis. Cell cycle arrest was also observed in HCC cells when they were treated with inhibitors of PI3K. For example, the treatment of Huh7 and HepG2 cells with copanlisib induced cell-cycle arrest via the downregulation of CDK4/6 and cyclin D1, although this treatment had a minor effect on apoptosis [48].

MK2206 effectively interacts with the pleckstrin–homology (PH) domain of AKT and hinders its recruitment in the plasma membrane, inhibiting PDK1 binding and the subsequent activation of AKT [54]. MK2206 has shown strong potency in inhibiting AKT [55]. Similar to the findings in HCC cells treated with PI3K inhibitors, treatment with MK2206 also induced growth inhibition and apoptosis in HCC cells [50,51]. The AKT Inhibitor VIII, which also blocks the activity of AKT through binding to the pleckstrin homology (PH) domain in AKT, suppressed cell growth and induced apoptosis in HCC cells [46]. 

Recent studies have indicated that mTOR plays a critical role in maintaining stemness-related functions in cancer stem cells (CSCs), and the inhibition of mTOR leads to the sensitization of CSCs to radiation therapy in breast cancer [56]. In line with these findings, treatment with rapamycin, the prototypic mTOR inhibitor, significantly reduces the frequency of CD133+/EpCAM+ cells in Hep3B and Huh7, which are widely considered liver cancer stem cell populations [52]. RAD001, also known as everolimus, is an inhibitor of mTOR. Its binding to the FK506-binding protein12 (FKBP12) allows the RAD001–FKBP12 complex to interact with mTOR, which subsequently inhibits S6K1 and 4EBP1 phosphorylation by mTOR. Treatment with RAD001 resulted in the induction of apoptosis [50], as well as a decrease in cell proliferation in diverse HCC cell lines, including Hep3B, Huh7, and HepG2 [50,51]. 

The PI3K/mTOR dual inhibitor BEZ235 induced growth inhibition and apoptosis in HCC cells [50]. Similarly, the treatment of HCC cells with Lenvatinib, which targets both AKT and mTOR, exhibited inhibitory effects on cell proliferation and migration [53]. In summary, targeting the EGFR/PI3K/AKT/mTOR signaling pathway via the inhibition of EGFR, PI3K, AKT, and/or mTOR has consistently shown anti-tumor effects on HCC cells in vitro (Table 1).

### 3.3. Targeting EGFR/PI3K/AKT/mTOR Signaling on Sorafenib-Resistant HCC Cells 

Sorafenib is the first-line systemic therapeutic for patients with advanced HCC, which inhibits both receptor tyrosine kinases (RTKs) and RAF [57]. However, the development of resistance to drugs and disease progression are nearly inevitable during the course of treatment [58]. Sorafenib resistance appears to be associated with the activation of PI3K/AKT/mTOR signaling [59]. Therefore, the combination of sorafenib and an inhibitor of the EGFR/PI3K/AKT/mTOR signaling pathway has been proposed as an effective therapeutic approach [45,51,60].

Copanlisib, a PI3K inhibitor, down-regulates downstream targets of AKT, leading to cell-cycle arrest and the suppression of cell proliferation, although it has a minimal effect on apoptosis [48,61]. Copanlisib counteracted sorafenib-induced AKT phosphorylation and synergistically enhanced anti-tumor effects on HCC when combined with sorafenib [48]. Likewise, combined treatment with sorafenib and capsaicin, an inhibitor of the PI3K/AKT/mTOR signaling pathway, also showed enhanced anti-tumor effects in Hep3B and HuH7 cells [62].

Lenvatinib is another first-line treatment for HCC which also inhibits RTKs. The combination of lenvatinib and copanlisib effectively suppressed the phosphorylation of AKT, which had been induced by treatment with lenvatinib. Copanlisib enhanced pro-apoptotic effects on HCC cell lines that were resistant to Lenvatinib [45]. Moreover, the sequential treatment of Huh7 cells with rapamycin, an mTOR inhibitor, following treatment with sorafenib substantially increased the sensitivity of HCC cells to sorafenib and decreased the frequency of liver cancer stem cell (CSC)-like cells, which are considered primary cells resistant to chemotherapy [52].

In summary, the combination of sorafenib or lenvatinib with agents targeting PI3K/AKT/mTOR signaling can enhance the anticancer activity of RTK inhibitors and is expected to overcome the emergence of therapy-resistant cells.

## 4. Animal Studies Investigating EGFR/PI3K/AKT/mTOR Signaling in HCC

### 4.1. Animal Models for HCC Induced by Activated EGFR/PI3K/AKT/mTOR Signaling

Studies have demonstrated the induction of hepatocarcinogenesis through the modification of genes involved in the PI3K/AKT/mTOR signaling pathway (Table 2). PTEN functions as a negative regulator of PI3K/AKT/mTOR signaling because it counteracts PI3K-mediated AKT activation (Figure 1). For the inactivation of PTEN, specifically in the liver, genetically engineered mice were used that carried two *Pten* alleles flanked by loxP sites as well as the *Cre* transgene under the promoter of albumin. These mice (referred to as “AlbCre; *Pten^fl/fl^* mice”) were prone to the development of HCC as well as intrahepatic cholangiocarcinoma (ICC). Tumors from the mice exhibited significant increases in the phosphorylated level of AKT, confirming the activation of the PI3K/AKT/mTOR signaling in *Pten*-deleted livers [63]. 

Recently, simple liver-specific transgenesis has been developed that allows the liver in adult mice to be transfected with DNA. This methodology, called hydrodynamic tail vein injection or simply HTVI, employs the application of physical force through the rapid injection of a large volume of DNA solution into the lateral tail vein. This process generates increased pressure within the vena cava, propelling the DNA solution into the large hepatic vein and eventually reaching the hepatic tissue and hepatocytes [69,70]. For genome editing in the liver using the CRISPR/Cas system, plasmids containing Cas9 and sgRNA are delivered to the liver via the HTVI method [71]. Using CRISPR-based gene editing combined with the HTVI method, the *Pten* gene was ablated in a subset of hepatocytes in murine livers [64]. In this setting, however, the tumor failed to develop in mice (referred to as “sgPten mice”). The discrepancy in these results between sgPten and AlbCre; *Pten^fl/fl^* mice were thought to be partially due to the lower frequency of hepatocytes having undergone the loss of PTEN in sgPten mice compared with that in AlbCre; *Pten^fl/fl^* mice. Moreover, in the case of the latter, the *Pten* gene was deleted during early embryogenesis, as opposed to sgPten mice, in which the deletion was induced in adult livers. Considering massive cell divisions during embryonic and fetal development, the deletion of *Pten* in embryos is expected to create a more favorable tissue environment for hepatocarcinogenesis [72]. Although the tumor failed to develop in the sgPten mice, concomitant c-Met overexpression in the liver led to the formation of hepatic adenomas (HCA) as well as HCC [64].

Plasmids delivered to the liver via HTVI remain as episomes in the nucleus and, thus, are prone to degradation. To overcome this limitation, the *Sleeping Beauty* transposon system is often coupled with HTVI, which allows genes of interest to be integrated into the genome. The HTVI of transposons encoding an activated form of AKT (myr-AKT1) alone induced HCC after 24 weeks post-HTVI [65,66]. Notably, the simultaneous expression of myr-AKT1 and c-Met significantly accelerated HCC development, causing complete lethality by 8 weeks post-HTVI [65]. Likewise, the co-expression of myr-AKT1 and N-RasG12V led to the rapid emergence of HCC [66]. The studies indicate that oncogenic AKT synergistically cooperated with another oncogene in the development of HCC. 

TSC1 is an upstream inhibitor of mTOR which suppresses an mTOR activator, RHEB (Figure 1). The liver-specific knockout of *Tsc1* using a similar method, as described in AlbCre; *Pten^fl/fl^* mice, resulted in the development of HCC by the age of 9–10 months old [67]. Similar experiments using AlbCre; *Tsc1^fl/f^* mice conducted by other groups also showed HCC development with a minor presence of ICC by 40 weeks of age. Tumors from AlbCre; *Tsc1^fl/f^* mice consistently exhibited the activation of mTOR signaling [63,68]. Of note, the concomitant deletion of both *Tsc1* and *Pten* in the liver gave rise to the rapid induction of HCC, usually around the age of 14 weeks, showing the simultaneous deletion of the two major negative regulators of PI3K/AKT/mTOR signaling strongly enhanced the signaling pathway and carcinogenesis in the liver [63].

### 4.2. Preclinical Animal Studies Targeting EGFR/PI3K/AKT/mTOR Signaling in HCC

Since the activation of the EGFR/PI3K/AKT/mTOR signaling pathway significantly contributes to HCC development, various inhibitors of this signaling pathway have been administered to murine models of HCC to investigate their effects on HCC in vivo (Table 3). MUC15 is a member of the high-molecular-weight glycoprotein family of Mucins. It has been shown to bind to EGFR and induce EGFR degradation, subsequently suppressing the downstream PI3K/AKT/mTOR signaling pathway [73]. In xenograft models of HCC, mice transplanted with HCCLM3 cells overexpressing MUC15 displayed fewer and smaller tumors, leading to increased survival rates compared to the control mice transplanted with HCC cells overexpressing the green fluorescent protein (GFP): an inert protein. Additionally, the degradation of EGFR by MUC15 resulted in fewer lung metastases in the MUC15 group compared to the control [73]. RHEB, as an activator of mTOR, was also targeted in a xenograft model of HCC. When human HCC cells, which were manipulated to express short hairpin RNA downregulating RHEB (RHEB-shRNA), were transplanted to immune-deficient mice, they grew more slowly than tumor cells expressing the control of shRNA in vivo [74].

The pharmacological inhibition of PI3K, AKT, or mTOR has exerted similar anti-cancer effects in xenograft models of HCC. Recently, a novel small-molecule inhibitor of PI3K, DZW-310, was developed. The administration of the drug to a Hep3B xenograft tumor model resulted in a significant decrease in tumor growth [75]. As observed in in vitro studies using HCC cells (see above in Section 3.2), administration with an AKT inhibitor, MK2206, led to the growth inhibition of tumors in xenograft models transplanted with Hep3B and Huh7 [50]. A novel dual inhibitor was developed that simultaneously inhibited AKT and mTOR. The compound, ZJQ-24, suppressed tumor growth in HepG2 xenograft mice in a dose-dependent manner [76]. 

Rapamycin (sirolimus) and its derivatives, RAD001 (also known as everolimus), function as highly selective allosteric inhibitors of mTORC1. They bind tightly to its primary target, the FK506-binding protein12 (FKBP12), which allows the complex to associate with the FKBP-rapamycin binding domain (FRB domain) of mTOR, which is located proximal to the active site of mTOR kinase. The formation of the FKBP12–rapamycin–mTOR complex restricts the access of mTOR substrates and, thus, inhibits the phosphorylation and activation of S6K1 and 4EBP1 [82,83]. In patient-derived HCC xenograft models, treatment with RAD001 led to a significant reduction in tumor growth in a dose-dependent manner [77]. Molecular analysis confirmed significant decreases in the phosphorylated levels of S6K1 and 4EBP1, while the level of phosphorylated mTOR was not altered. RAD001-induced growth suppression was associated with the inhibition of cell proliferation via the downregulation of Cdk-6, Cdk-2, Cdk-4, cdc-25C, cyclin B1 and c-Myc [77]. Treatment with rapamycin alone in the HepG2 xenograft model, however, showed no significant benefit to animal survival compared to the control mice treated with the vehicle [78]. Instead, combination therapy with rapamycin and bevacizumab, a monoclonal antibody targeting the vascular endothelial growth factor (VEGF), led to reduced tumor sizes as well as prolonged survival in xenograft mice. Although the discrepancy in the results between RAD001 and rapamycin treatments was not clear, it was speculated that xenograft mice transplanted with HCC directly derived from patients might better represent the characteristics of human HCC compared to those transplanted with HepG2, which had been long maintained in the tissue culture in vitro. Metformin inhibits mTOR via the activation of AMPK: a strong suppressor of mTOR. It is noteworthy that treatment with metformin also retarded tumor development in HCC xenograft models transplanted with Bel-7402 cells [79].

Recently, there has been increasing interest in applying the RNA interference (RNAi) approach to HCC therapy [84,85,86]. A variety of short interfering RNAs (siRNAs), as well as miRNAs have been tested for anti-cancer effects in HCC cells in vitro, targeting the major components of the EGFR/PI3K/AKT/mTOR signaling pathway. For example, miRNAs targeting EGFR, such as miRNA-137, miRNA-302b, and miRNA-486-3p, have consistently shown anti-proliferative effects on HCC cells, including HepG2, Huh7, and SMMC-7721 [87,88,89]. Likewise, PI3K and AKT were downregulated in HepG2 cells using miRNA-124 and miRNA-149, respectively, which led to decreases in the proliferation and migration of HCC cells [90,91]. In particular, attempts have been made to inhibit mTOR using miRNA in vivo. The intravenous injection of miRNA-199a-3p encapsulated in nanoparticles efficiently reduced the growth of tumors in HCC xenograft mice [80]. In this study, target delivery to the liver was achieved by conjugating lactobionic acid (LA) with nanoparticles, the receptor of which was overexpressed in HCC cells. In another study, the intratumoral injection of cholesterol-conjugated miR-99a, which targets mTOR, led to a reduction in tumor mass in xenograft mice bearing HCC [81]. With the recent improvements in the stability of RNA, nanoparticle technology, and targeted delivery, RNAi-based therapy is expected to arise as a promising approach to target the EGFR/PI3K/AKT/mTOR signaling specifically and effectively in HCC in vivo.

## 5. Clinical Studies Targeting EGFR/PI3K/AKT/mTOR Signaling 

Given the significant roles of the EGFR/PI3K/AKT/mTOR pathway in human cancer, there has been a strong emphasis on investigating its clinical potential by targeting the major components of the EGFR/PI3K/AKT/mTOR signaling pathway using specific small-molecule inhibitors. For instance, copanlisib, an inhibitor specific to PI3K, was administered to patients with cancers carrying an activating mutation in PI3K (NCT02465060) [92]. In a similar vein, a phase II clinical trial evaluated the efficacy of the allosteric AKT inhibitor MK-2206 (NCT01239355) [93]. Both clinical studies, however, were prematurely terminated due to discouraging results, including various adverse side effects (Table 4). 

Various drugs have been employed to inhibit mTOR in clinical studies of HCC [94]. In the phase I/II study, patients with advanced HCC were given everolimus at 10 mg/day as a single agent. The treatment failed to consistently show effectiveness, as only two patients (8%) were progression-free at 24 weeks (NCT00516165). HCC patients treated with the mTOR inhibitor sirolimus (rapamycin) exhibited significantly longer overall survival (OS) compared to the control group [95]. A phase II clinical study demonstrated that sirolimus extended the survival of patients with HCC after liver transplantation (NCT01374750). Onatasertib (CC-223), an orally administered mTOR inhibitor, has been found to induce mitochondrial dysfunction in HCC cell lines [96]. In an open-label phase II trial (NCT03591965), researchers explored the use of onatasertib in subjects with hepatitis B virus (HBV)-positive HCC who had previously undergone at least one line of therapy. Another completed phase I/II study investigated AZD8055, a novel ATP-competitive mTOR kinase inhibitor, evaluating its safety, tolerability, pharmacokinetics, and preliminary efficacy [97]. Overall, although some clinical trials have shown that mTOR inhibitors can provide a survival benefit in patients with advanced HCC, especially when they were intolerant to sorafenib (a standard first-line therapy for advanced HCC), not all trials have demonstrated a significant improvement in overall survival with the treatment of an mTOR inhibitor.

The combinatory inhibition of mTOR and VEGF using rapamycin and bevacizumab significantly reduced tumor growth in xenograft models of HCC [78] (Table 3). A phase I clinical trial explored the combination of rapamycin with bevacizumab at the maximum tolerated dose in patients with HCC, demonstrating a complete response in one case and stable disease states in the majority, although this combinatory treatment did not show significant improvements in overall survival compared to patients treated with bevacizumab only (NCT00467194). Phase I/II clinical trials are ongoing to further investigate the efficacy of the combination therapy in HCC [94]. Erlotinib is an EGFR inhibitor that effectively suppresses the phosphorylation of its downstream effectors, such as AKT and mTOR. The combined inhibition of EGFR and VEGF using erlotinib and bevacizumab showed similar anti-cancer effects in patients with advanced HCC (NCT00881751) [98].

In an attempt to improve outcomes, a single-arm phase II trial combined temsirolimus and sorafenib to exploit mTOR inhibition along with sorafenib’s effects on HCC [99]. However, it failed to achieve an improvement in overall survival compared to the control group, who were treated with sorafenib only (NCT01687673). Despite this setback, ongoing research continues to explore drug combinations, and several clinical studies are underway [100,101]. It is noteworthy that while mTOR inhibitors, as well as other therapeutics targeting the EGFR/PI3K/AKT/mTOR pathway, have shown some promise in preclinical studies, their efficacy in patients with advanced HCC remains uncertain. These findings highlight the complexity of targeting the EGFR/PI3K/AKT/mTOR pathway in HCC and underscore the need for further investigation to identify more effective treatment approaches. 

## 6. Perspectives and Conclusions

A comprehensive understanding of the molecular pathway leading to carcinogenesis is crucial for predicting patient responses to targeted therapies, significantly impacting clinical decision-making. The development of HCC is a complex process involving various alterations in multiple signaling cascades [5,6]. Among the various oncogenic signals, the EGFR/PI3K/AKT/mTOR pathway stands out as it is activated in over 50% of HCC cases, making it a crucial target for patients with this condition. Research has emphasized the pivotal role of the EGFR/PI3K/AKT/mTOR cascade in the development of HCC. Promisingly, preclinical studies using human HCC cells have consistently demonstrated the effectiveness of targeting the key components of this signaling pathway. Such interventions have been shown to suppress the proliferation of tumor cells in vitro and inhibit the growth of HCC in vivo, offering hope for potential therapeutic approaches. 

While preclinical studies have shown promise in targeting the PI3K/AKT/mTOR pathway, clinical trials have not consistently demonstrated a significant improvement in patient outcomes. The overall response rate and survival benefits observed in clinical studies targeting the EGFR/PI3K/AKT/mTOR signaling pathway have been modest or disappointing. These less-than-satisfactory outcomes could be attributed, in part, to the significant toxicities and adverse effects caused by PI3K/AKT/mTOR inhibitors, negatively impacting patients’ quality of life and leading to treatment discontinuation. Notably, adverse effects routinely observed in administering these inhibitors include decreased liver functions, as determined by increased alkaline phosphatase (ALP) and aspartate aminotransferase (AST) levels, hypoalbuminemia, thrombocytopenia, and hyperbilirubinemia (Table 4). The side effects on the liver exerted by PI3K/AKT/mTOR inhibitors are especially detrimental to HCC patients who already have pre-existing liver damage. 

In this sense, successful therapy for HCC should enable the efficient targeting of the EGFR/PI3K/AKT/mTOR pathway with minimal toxicities and adverse effects. One promising approach is the targeted delivery of drugs to HCC using polymeric nanoparticles (PNPs) [86,102]. In other words, for HCC-targeted delivery, inhibitors of EGFR/PI3K/AKT/mTOR signaling are encapsulated in PNPs that are conjugated with targeting ligands specific to HCC cells. For example, the asialoglycoprotein receptors (ASGP-R) and Glypican-3 (GPC3) are overexpressed in HCC and are considered highly specific markers for HCC [103,104]. Conjugating monoclonal antibodies or ligands that specifically recognize ASGP-R or GPC3 with PNPs are expected to allow the encapsulated drugs to be specifically delivered to HCC, minimizing various side effects and drug-related toxicities commonly found in the systemic delivery of these inhibitors for EGFR/PI3K/AKT/mTOR signaling. Thus, there is hope for the future as new drugs and therapeutic approaches are anticipated to be developed.

## Figures and Tables

**Table 1:** The inhibition of the EGFR/PI3K/AKT/mTOR pathway in HCC cell lines.

Drug	Target	HCC Cell Line	Phenotype	Reference
erlotinib	EGFR	MHCC97H, PLC/RLF/5	reduced cell proliferation	[32]
		Huh7	reduced migration	[44]
AG1478	EGFR	HepG2	reduced cell proliferation and invasion	[38]
gefitinib	EGFR	Huh7	induced apoptosis	[45]
GW2974	EGFR	HepG2	reduced cell proliferation, induced apoptosis	[41]
copanlisib	PI3K	Huh7, HepG2	induced apoptosis, inhibited cell growth, inducing cell cycle arrest	[45,48]
LY294002	PI3K	MHCC97-H	induced apoptosis	[49]
		Huh7, Mahlavu	suppressed cell growth, induced apoptosis	[46]
wortmannin	PI3K	Huh7, Mahlavu	suppressed cell growth, induced apoptosis	[46]
		HepG2	induced apoptosis	[46]
740Y-P	PI3K	SMMC-7721, MHCC-97H	reduced cell proliferation, induced apoptosis	[47]
MK2206	AKT	Hep3B, Huh7, PLC/RLF/5	growth-inhibitory, induced apoptosis	[50]
		HepG2	anti-proliferative	[51]
AKT inhibitor VIII	AKT	Huh7, Mahlavu	suppressed cell growth, induced apoptosis	[46]
rapamycin	mTOR	Hep3B	prevent enrichment of CSCs	[52]
RAD001 (everolimus)	mTOR	Hep3B, Huh7, PLC/RLF/5	growth-inhibitory, induced apoptosis	[50]
BEZ235	PI3K/mTOR	Hep3B, Huh7, PLC/RLF/5	growth-inhibitory, induced apoptosis	[50]
lenvatinib	AKT/mTOR	Hep3B, HepG2	reduced cell proliferation, migration	[53]

**Table 2:** Mouse models for HCC induced by activated EGFR/PI3K/AKT/mTOR signaling.

Gene	Mouse Model	Phenotype/Tumor Type	Ref.
*Pten*	Alb-Cre; *Pten^fl/fl^*	ballooning, steatosis/ICC, HCC	[63]
	sgPten sgPten/c-Met	lipid accumulation/no tumor lipid accumulation/HCA, HCC	[64]
*AKT*	HA-myr-AKT1 HA-myr-AKT1/V5-c-Met	hepatic steatosis, proliferation/HCC rapid liver tumor growth	[65]
	myr-AKT1 myr-AKT1/N-RasG12V	hepatocyte proliferation/HCA (12 w), HCC (6 m) spotty and pale color/nodular lesions (~4 w)	[66]
*Tsc1/Tsc2*	Alb-Cre; *Tsc1^fl/fl^*	dysplastic foci, nodules, hepatomas/HCC	[67]
	Alb-Cre; *Tsc1^fl/fl^*	moderate tumor incidence rate/HCC	[68]
	Alb-Cre; *Tsc1^fl/fl^* Alb-Cre; *Tsc1^fl/fl^*/*Pten^fl/fl^*	no steatosis/HCC, ICC hepatomegaly (early), large tumor (14 w)/HCC	[63]

ICC, intrahepatic cholangiocarcinoma; HCA, hepatocellular adenoma; HCC, hepatocellular carcinoma.

**Table 3:** HCC suppression in xenograft models by targeting EGFR/PI3K/AKT/mTOR signaling.

Agent	Target	Administration Route	HCC Cell Line Transplanted	Phenotype	Ref.
MUC15	EGFR	NA	HCCLM3	delayed tumor formation, higher survival rate	[73]
RHEB-shRNA	RHEB	NA	SMMC-7721	decrease in tumor growth	[74]
DZW-310	PI3K	OA	Hep3B	decrease in tumor growth	[75]
MK2206	AKT	IP	Hep3B, Huh7	decrease in tumor growth	[50]
ZJQ-24	AKT/mTOR	OA	HepG2	Tumor cell death/reduced tumor growth	[76]
RAD001	mTOR	OA	Patient-derived HCCs	decrease in tumor growth	[77]
rapamycin	mTOR	OA	HepG2	no effects	[78]
rapamycin + bevacizumab	mTOR + VEGF	OA + IP	HepG2	decreased tumor size/increased survival	[78]
metformin	mTOR	IP	Bel-7402	decrease in tumor growth	[79]
miRNA-199a-3p	mTOR	IV	Huh7	decreased tumor growth/increased survival	[80]
miRNA-99a	mTOR	IT	SMMC-LTNM	reduction in tumor size	[81]

NA, not applicable; OA, oral administration; IP, intraperitoneal; IV, intravenous; IT, intertumoral.

**Table 4:** Clinical trials targeting the EGFR/PI3K/Akt/mTOR signaling pathway in HCC.

Agent	Target	Phase	Clinical Outcomes	Adverse Events (Side Effects)	NCT #
Copanlisib	pan-class I PI3K	II	discontinuation due to disease progression	hyperglycemia (63%), fatigue (40%), diarrhea (37%), hypertension (33%), nausea (33%), maculopapular rash (30%)	02465060
MK-2206	AKT	II	early termination for discouraging results	anemia (73.33%), hyperglycemia (60.00%), hypoalbuminemia (46.67%), hyperbilirubinaemia (13.33%)	01239355
everolimus (RAD001)	mTOR	I/II	only 2 patients (8%) responded to treatment	hyperglycemia (42.86%), diarrhea (39.29%), hyponatremia (32.14%)	00516165
sirolimus (rapamycin)	mTOR	II	MOS of 21.1 m vs. 14.1 m for control (survival benefit over control)	dyslipidaemia, oral mucositis, diarrhea	01374750
onatasertib (CC-223)	mTOR	II	preliminary antitumor activity	diarrhea (60.38%), hyperglycaemia (60.38%), thrombocytopenia (30.19%), hyperbilirubinaemia (11.32%)	03591965
AZD8055	mTOR	I/II	not yet determined	increased aspartate aminotransferase (22%), fatigue (16%)	00999882
rapamycin + bevacizumab	mTOR + VEGF	I	no survival benefit over bevacizumab-only treatment	hyperglycaemia (83%), thrombocytopenia (75%), fatigue (46%), mucositis (46%), anorexia (42%), Diarrhea (33%)	00467194
erlotinib + bevacizumab	EGFR + VEGF	II	no improvement over sorafenib-treated group	increased alkaline phosphatase (38.30%), hypoalbuminemia (29.79%)	00881751
temsirolimus + sorafenib	mTOR + RTK	II	no survival benefit over sorafenib-only group	hypophosphatemia (60.71%), diarrhea (28.57%), anemia (10.71%)	01687673

MOS, mean overall survival; VEGF, vascular endothelial growth factor; RTK, receptor tyrosine kinase.

## Data Availability

Not applicable.
